# Visualization of ribosomal RNA operon copy number distribution

**DOI:** 10.1186/1471-2180-9-208

**Published:** 2009-09-25

**Authors:** Rajat Rastogi, Martin Wu, Indrani DasGupta, George E Fox

**Affiliations:** 1 Dept of Computer Science, University of Houston, Houston, Texas, USA; 2 Dept of Biology, University of Virginia, Charlottesville, Virginia, USA; 3 Dept of Biology and Biochemistry, University of Houston, Houston, Texas, USA

## Abstract

**Background:**

Results of microbial ecology studies using 16S rRNA sequence information can be deceiving due to differences in rRNA operon copy number and genome size of the detected organisms. It therefore will be useful for investigators to have a better understanding of how these two parameters differ in various organism types. In this study, the number of ribosomal operons and genome size were separately mapped onto a Bacterial phylogenetic tree.

**Results:**

A representative Bacterial tree was constructed using 31 marker genes found in 578 bacterial genome sequences. Organism names are displayed on the trees using graduations of color such that similar colors indicate similar numbers of operons or genome size. The resulting images provide an intuitive understanding of how copy number and genome size vary in different Bacterial phyla.

**Conclusion:**

Once the phylogenetic position of a novel organism is known the number of rRNA operons, and to a lesser extent the genome size, can be estimated by examination of the colored maps. Further detail can then be obtained for members of relevant taxa from the rrnDB database.

## Background

The ribosomal RNA (rRNA) genes of Bacteria and Archaea are typically found in operons. Although many organisms have a single rRNA operon the actual number is known to vary between 1 and 15 [1]. The operons themselves do not always exhibit the same sequence but instead different in a modest number of positions, typically less than 15 in the case of 16S rRNAs. Nevertheless, there are exceptions. For example, one of the three 16S rRNA genes in *Halobacterium marismortui *differs from the others in over 70 positions [2]. Such microheterogeneity has been studied in detail in a modest number of cases. For example, it has been recently shown is in *Streptomyces coelicolor *that all the operons are expressed and their RNAs incorporated into ribosomes but the relative expression level may vary over the growth cycle [3,4]. In the case of *H. marismortui*, the aberrant operon responds to temperature differently [5]. Efforts to evaluate the extent of rRNA operon microheterogeneity likely should be handled cautiously. An examination of complete genome sequences revealed many examples where all the 16S rRNA genes in an organism with multiple rRNA operons are reported to be identical [6]. There certainly are cases where multiple rRNAs exist with the same sequence. However, in the case of the rapidly accumulating bacterial genomes, one must remember that long nearly exactly repeated regions are difficult to sequence. Thus, one must consider the possibility that at least some and perhaps many, of the assembled genomes are reporting multiple copies of what are actually consensus rRNA sequences.

Although the true extent of microheterogeneity may be underestimated in the published genomes, the numbers of operons present is likely reliable. Since 2001 the number of ribosomal operons has been curated in the rrnDB (Ribosomal RNA Operon Copy Number Database) [7,8] for all instances where it is known. The number of rRNA operons is believed to in part be correlated with organism ecological strategy [9-11]. Operon number is of special interest when 16S rRNA sequence information is used to study the composition of microbial ecosystems because organisms with larger numbers of copies of the rRNA operon will be disproportionately represented in the resulting profiles [12]. Therefore, when attempting to quantify relative numbers in environmental populations, it is appropriate to correct the data by taking into account both the genome size and the number of operons [13]. However, this is potentially problematic as many of the strains that are encountered have no exact match in the database and it is therefore not immediately apparent how many operons are likely to be present or what the genome size is likely to be. Herein, we examine this issue by mapping these two traits onto a phylogenetic tree [14]. Once one determines the approximate phylogenetic position of an organism one can use these maps to make a reasonable assessment of genome size and especially, rRNA operon copy number.

## Methods

### Tree Construction

Homologs of each of the 31 phylogenetic marker genes(dnaG, frr, infC, nusA, pgk, pyrG, rplA, rplB, rplC, rplD, rplE, rplF, rplK, rplL, rplM, rplN, rplP, rplS, rplT, rpmA, rpoB, rpsB, rpsC, rpsE, rpsI, rpsJ, rpsK, rpsM, rpsS, smpB, tsf) were identified from the 578 bacterial genomes that were complete at the time of the study. The corresponding protein sequences were retrieved, aligned, and trimmed and then concatenated by species into a mega-alignment [15]. A maximum likelihood tree was then constructed from the mega-alignment using PHYML. The model selected based on the likelihood ratio test was the Whelan and Goldman (WAG) model of amino acid substitution with gamma-distributed rate variation (5 categories) and a proportion of invariable sites. The shape of the gamma-distribution and the proportion of the invariable sites were estimated by the program

### Tree Labeling

The number of ribosomal operons in each genome and the size of the genome were obtained from the NCBI website http://www.ncbi.nlm.nih.gov/genomes/lproks.cgi. In a small number of instances bacteria are considered to have multiple chromosomes. In these cases, the total number of operons in all the chromosomes was used and the combined mass of the multiple chromosomes used for genome size. In addition, in some instances the number of copies of each rRNA is different. This is most frequent for 5S rRNA, which may be present in an extra copy. In these cases, the number of 16S rRNA genes was used as the number of operons as in most practical applications it is 16S rRNA that is being examined. The tree was combined with the operon and information and built using Newick format such that each node is specified http://en.wikipedia.org/wiki/Newick by "species-name*genome-size*rRNA-operon-count". The organism names on the tree were colored according to either operon number or genome size. In each case, as the parameter increases the color generally becomes darker. Thus, for the operons 14 colors were used. For 0 to 6 operons, shades of yellow, orange or red were used with darker colors indicating larger numbers of operons. For 7 to 10 operons shades of blue were used and greens were used for 11 or more. In the case of genome size, 12 colors were used to depict various size ranges. The first range was 0-1 MB with subsequent increments of 0.5 MB. The final range was for genomes greater than 6 MB in size. The final tree was created in the .esp format using ATV [16].

## Results

**Figure 1:**
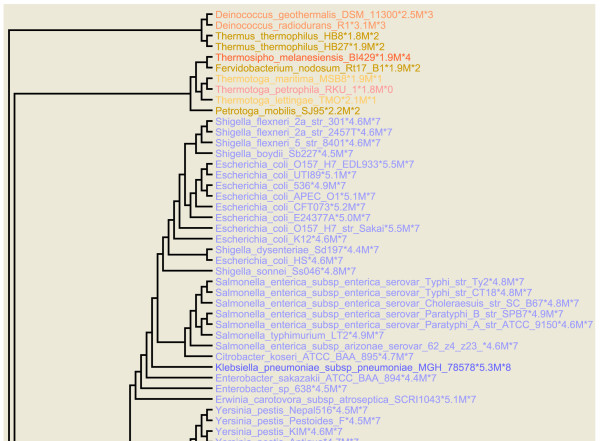
**Phylogenetic tree colored according to operon copy number**. Each organism name on the tree is followed by the approximate size of its genome in megabases, (MB), and the number of rRNA operons found in the genome. The color of the lettering is decided by the number of operons. Fourteen distinct colors were used with each assigned to a specific number of operons. As the operon number increases the color used generally becomes darker. The darkish shade of green is used for 13 or more copies. This figure shows the upper quartile, for the full image please see Additional file 1.

**Figure 2:**
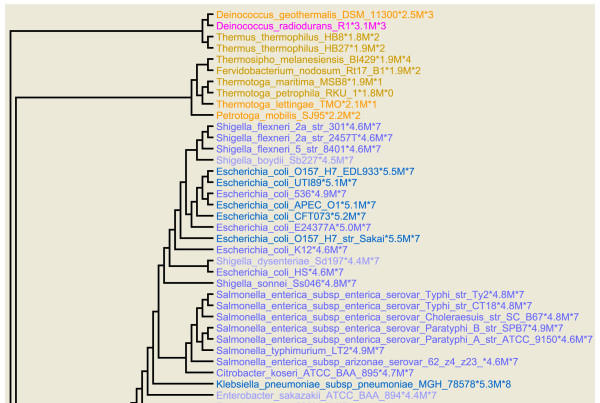
**Phylogenetic tree colored according to genome size**. Each organism name on the tree is followed by the approximate size of its genome in megabases and the number of rRNA operons found in the genome. The color of the lettering is decided by the size of the genome. Twelve distinct colors were used with each assigned to a genome size range. The lightest color was used for genomes up to 1 MB. Subsequently, colors were assigned to genome size ranges in increments of 0.5 MB. Genomes larger than 6 MB were all colored green. This figure shows the upper quartile, for the full image please see Additional file 2.

Bacterial rRNA operon copy number was mapped onto a phylogenetic tree by coloring the organism names on each branch in accordance with the number of operons (Figure 1 and Additional file 1). Genome size was separately mapped in a similar manner (Figure 2 and Additional file 2). These maps allow one to readily visualize the extent to which these properties have been conserved over phylogenetic distance. In both cases, the values are conserved within species and frequently within genera as well. In the case of operon number, similar values are frequently found in neighboring groupings as well. Overall, rRNA operon number typically only exceeds six in two regions of the tree, the γ-Proteobacteria and the Firmicutes, e.g. *Bacillus*, *Staphylococcus*, *Streptococcus*, and others [8]. Thus, if one knows the approximate phylogenetic position of an organism one can make a reasonable prediction of how many rRNA operons it will have. As previously noted, genome size and operon number are largely uncorrelated with the one exception that organisms with genome sizes below 1.5 MB almost never have more than one rRNA operon.

**Figure 3:**
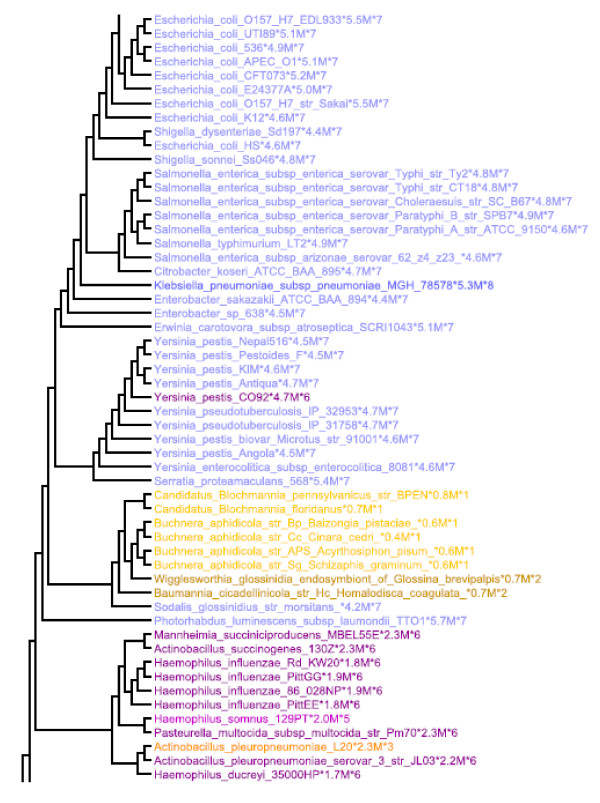
**Excerpt from Figure 1 showing a portion of the γ-Proteobacteria as discussed in the text**. Coloring is as in Figure 1.

These observations are illustrated in Figure 3, which is excerpted from Figure 1 and shows a portion of the γ-Proteobacteria. Here one sees that for a large number of enterics (*Escherichia, Salmonella*, *Yersinia *etc) the operon number is typically seven with only occasional strains, having six or eight operons. Related genera such as *Mannheimia *and *Haemophilus *typically have 5 or 6 operons. However, *Candidatus biochmannia *and *Buchnera *strains have only one operon. The difference here is genome size. These organisms all have genomes less than 1 MB. The predictions are of course not perfect, and one will see occasional exceptions. Thus, in Figure 1, one *Actinobacillus *strain only has three operons while all of the other close neighbors have six.

## Discussion

The fact that members of the same species generally have essentially the same number of rRNA operons has been pointed out previously [6]. However, in the absence of the type of mapping shown here the phylogenetic extent to which this is true is not readily recognized. Initial mapping efforts [7] were not fully informative in this regard due to the modest number of species for which the requisite information was available at the time. Prior work has shown that rRNA copy number impacts organism life history [7,10]. This suggests that gain or loss of rRNA operons would appear to be a potential method of adapting to different environments and one might envision numerous individual organisms in populations as having different numbers of rRNA operon. Although rRNA operon copy number has typically not been examined in multiple individuals within a population, the high conservation of numbers within similar species from different sources argues against this.

The maps provided here will be especially useful to those seeking to quantitatively characterize microbial ecosystems using 16S rRNA sequence characterizations. The number of times an organism is encountered must be adjusted for the size of its genome and especially the number of copies of the 16S rRNA gene it carries. Once 16S rRNA sequence data is available the approximate phylogenetic position of each organism can be estimated. The mappings can then be examined to obtain initial estimates of rRNA operon number and genome size by examining the neighboring phylogenetic groupings. With the relevant phylogenetic groupings identified one can then use the rrnDB database [8] to obtain the values for all organisms belonging to those groups.

## Authors' contributions

GEF conceived of the study and wrote the paper. MW constructed the tree. ID and GEF tabulated the genome sizes and operon copy number data. RR drew the trees, devised and implemented the coloring schemes.

## Supplementary Material

Additional file 1 **Full image for Figure **1. Click here for file

Additional file 2 **Full image for Figure **2. Click here for file
