# Supplementary figures and images for: Visualization of ribosomal RNA operon copy number distribution
Source: BMC Microbiol. 2009 Sep 25;9:208. doi: 10.1186/1471-2180-9-208 (PMC2761929; doi:10.1186/1471-2180-9-208)

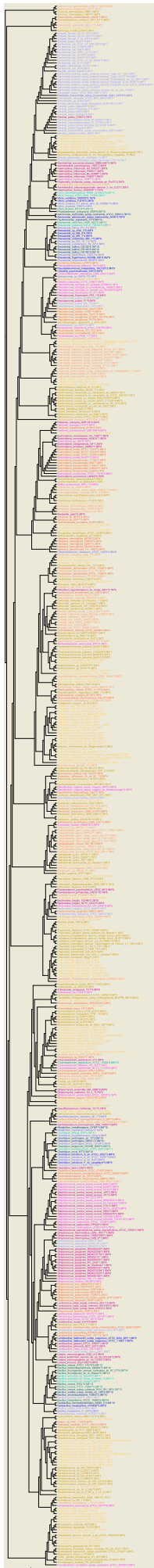

Supplement: Additional file 1 — Full image for Figure 1. [file 1471-2180-9-208-S1.PDF]

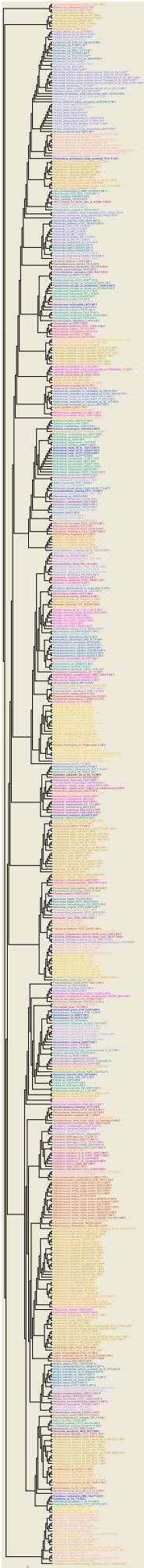

Supplement: Additional file 2 — Full image for Figure 2. [file 1471-2180-9-208-S2.PDF]
